# Incidental giant obstructed pedunculated gastric lipoma during gastrostomy: A case report

**DOI:** 10.1016/j.ijscr.2018.11.052

**Published:** 2018-11-24

**Authors:** Mohamad Emad Abdulrahman, Anas Aji, Mhd Belal Alsabek

**Affiliations:** a General Surgery Department, Damascus Hospital, P.O. Box: 8085, Damascus, Syria; b General Surgery Clinic, Baghdad Ave., Damascus, Syria; c General Surgery Department, Al-Mouwasat University Hospital, Mazzeh, Omar ben Abdulaziz St., Damascus, Syria; d Department of Surgery, Syrian Private University, Faculty of Medicine, Damascus, Syria

**Keywords:** Incidental gastric lipoma, Gastrostomy, Outlet gastric obstruction

## Abstract

**Background:**

Most gastric lipomas are small, asymptomatic, and detected as incidental findings on radiologic evaluation or endoscopic examination of the upper gastrointestinal tract. We report a large lipoma which was noticed by its mass effect during a percutaneous endoscopic gastrostomy; the full evaluation prevented an unexplained failed procedure.

**Presentation:**

A 65 year old man with a cerebrovascular accident (CVA) suffered from resistant vomiting and severe dysphagia. An endoscopic gastrostomy was indicated, but an incidental pedunculated submucosal mass in antrum terminated the procedure for further investigations, it was highly expected to cause an intermittent gastric outlet obstruction shortly after the procedure. The radiological evaluation found an 8 × 5 cm^2^ lipoma. A resectional surgery was performed and combined with an open gastrostomy tube placement.

**Discussion:**

Gastric lipoma is a very rare condition and mostly small and asymptomatic. Symptomatic ones could manifest by bleeding or obstruction. In our case, the obstruction was discovered incidentally by endoscopy. Computed tomography (CT) scan is an excellent diagnostic tool that confirmed the diagnosis. The treatment options range from open surgery to laparoscopic or endoscopic intervention.

**Conclusion:**

A full endoscopic evaluation for the upper gastrointestinal tract is recommended before gastrostomy. Our reported case gives an advantage of the percutaneous endoscopic gastrostomy procedure over the open gastrostomy tube placement to rule out a large asymptomatic gastric lipoma.

## Highlights

• Submucosal lipoma should be always considered in gastric obstruction.
• Endoscopic gastrostomy is better than surgical gastrostomy in excluding asymptomatic tumor.
• A full upper GI endoscopy is recommended before applying the gastrostomy.

## 1 Introduction

Gastrointestinal lipomas are tumors of mature adipose tissue, surrounded by fibrous capsule [1]. Gastric lipomas are very rare conditions that represent less than 5% of gastrointestinal lipomas and constitute less than 1% of all gastric neoplasms [2]. Most gastric lipomas are small, asymptomatic and mainly diagnosed by radiologic evaluation and endoscopic examination of the upper GI tract [3]. The most frequent clinical manifestation is GI bleeding (53%) which is due to ischemic ulceration of the overlying mucosa [4]. Other manifestations could be obstruction, abdominal pain or dyspepsia. Most lipomas are localized in submucosa layer (90%) and affect the antrum in 75% of cases [5,6]. In our case, a full upper GI endoscopy found an incidental giant lipoma which localized in the submucosa of antrum and caused unremarkable upper GI obstruction. The case has been reported in line with the SCARE criteria [7].

## 2 Case presentation

**Fig. 1:**
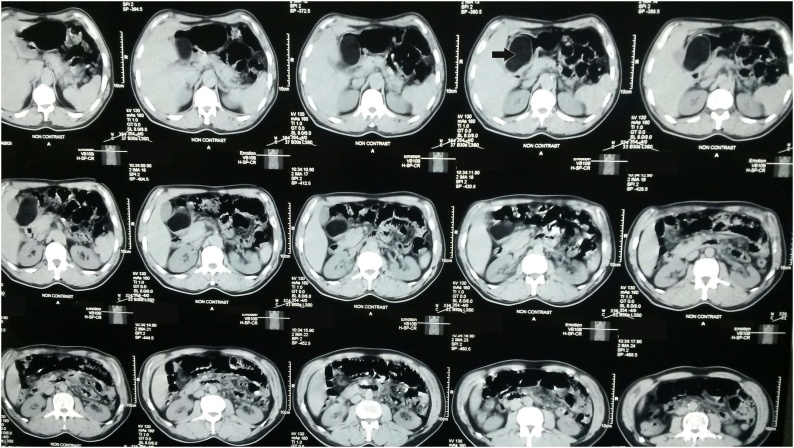
Abdominal CT shows a 4.5 × 8 cm^2^ submucosal encapsulated mass in the lower pyloric partition, fatty in its nature.

**Fig. 2:**
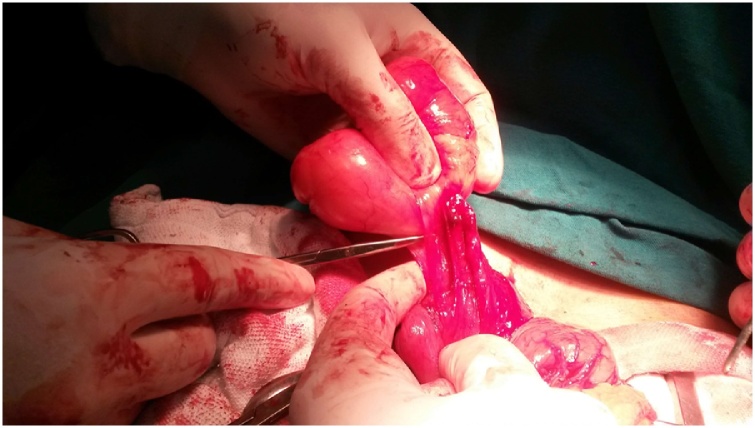
Intraluminal gastric tumor seen at the operative field arising from the submucosal layer of antrum.

A 65 year old man was referred to our clinic with a diagnosis of CVA, he developed multiple vomiting and sever dysphagia in every meal. The patient had been candidate to percutaneous endoscopic gastrostomy. Incidentally, the full upper endoscopy detected a gastric outlet obstruction; there was no intraluminal mass or ulceration on mucosa. This finding terminated the procedure without performing the gastrostomy. The patient underwent the full study, the CT scan of the abdomen showed a submucosal well encapsulated mass at the lower pyloric partition of stomach with a fatty like nature that measured 4.5 by 8 cm^2^ with no other remarkable findings [Fig. 1]. The patient was prepared for surgery. Open laparotomy was done; the mass was excised [Fig. 2] and a gastrostomy was established. The microscopical study stated that the lesion consisted of mature adipocytes without cytological atypia, arranged in lobules delimited by conjunctivovascular bays.

## 3 Discussion

Gastric lipoma is a very rare condition and mostly small and asymptomatic. Posterior wall of the antrum is the most site of origin [8]. Most cases are found in patients in their fifth and sixth decade with seemingly higher incidence in women than men [9]. The manifestations could be the results of bleeding due to mucosal ulceration over the tumor [10,11]. In other presentations when the tumor is large, pediculate and locating near the pylorus, it may cause a degree of obstruction [12]. Remarkably, these obstructive symptoms seem to be more prevalent in elderly male [13,14]. In our case, the obstruction was discovered incidentally by endoscopy as the vomiting started soon after the CVA, then the procedure has been terminated directly and CT scan administered. CT scan is an excellent investigation for GI tract lipoma that permits the specific diagnosis of lipoma based on fat density of the tumor, and precludes the need of endoscopic biopsy [15]. Homogeneous mass with fat density that is ranging between −70 and −120 HU is considered pathognomonic for the diagnosis of gastric lipoma [16]. Symptomatic gastric lipomas should be resected endoscopically or by open surgery. Endoscopic polypectomy can be tried for submucosal lesion smaller than 3 cm in diameter; larger broad based tumors have higher risks for perforation by endoscopic approach [17]. Surgical resection is still the main management of symptomatic large tumors. Laparoscopic resection has been advised for lipomas less than 6 cm in diameter in adults [18]. In our case, an incidental finding was managed by gastrostomy and enucleation. The mass was easily dissected and enucleated. We had satisfactory postoperative results and no vomiting had recorded in the weekly follow up visits.

## 4 Conclusion

Gastric lipoma is a rare condition mostly asymptomatic. However, it should be in mind when doing gastrostomy as it could fail the procedure and a resistant vomiting may be developed. We recommend a full endoscopic evaluation for the upper gastrointestinal tract whenever it is possible to rule out even a rare case of a large asymptomatic gastric lipoma.

## Conflicts of interest

There are no potential conflicts of interest.

## Funding

This research did not receive any specific grant from funding agencies in the public, commercial, or not-for-profit sectors.

## Ethical approval

This scientific paper is correspondent with the policies and ethics that are mentioned in the Elsevier guides.

## Consent

Written informed consent was obtained from the patient and is available upon request. No patient identifying material was used in this manuscript.

## Author contribution

Mohamad Emad Abdulrahman: corresponding Author, wrote the manuscript.

Anas Aji: the consultant who run the open surgery.

Mhd Belal Alsabek: supervisor, contribute writing the manuscript.

## Registration of research studies

This case report has not been registered because it is for a rare case with new plan of management.

It is not a research or a new surgical technique.

## Guarantor

Dr. Mhd Belal Alsabek.

## Provenance and peer review

Not commissioned, externally peer reviewed.
